# Low lean mass and all-cause mortality risk in the middle-aged and older population: a dose-response meta-analysis of prospective cohort studies

**DOI:** 10.3389/fmed.2025.1589888

**Published:** 2025-06-25

**Authors:** Juan Li, Xiaoling Liu, Qing Yang, Wenying Huang, Zhibin Nie, Yahai Wang

**Affiliations:** ^1^College of Arts and Physical Education, Nanchang Normal College of Applied Technology, Nanchang, China; ^2^Vascular Surgery, The Sixth Hospital of Wuhan, Affiliated Hospital of Jianghan University, Wuhan, China; ^3^College of Physical Education, Jiangxi Normal University, Nanchang, China

**Keywords:** lean mass, all-cause mortality, middle-aged and older population, meta-analysis, prospective cohort studies

## Abstract

**Objective:**

The accelerated aging process has raised substantial public health concerns regarding the health of the middle-aged and older population. The aim of our study was to investigate the association between low lean mass and the risk of all-cause mortality in older people, with the goal of promoting a long lifespan and reducing public health burdens.

**Methods:**

Three databases (PubMed, Web of Science, and Scopus) were searched for articles before May 22, 2025. The quality of the included articles was assessed using the Newcastle-Ottawa Scale (NOS). A meta-analysis was conducted using a random effects model. Subgroup analysis and meta-regression analysis were performed based on research characteristics. A dose-response analysis was performed to assess the specific association between lean mass and the risk of all-cause mortality. Sensitivity analysis was conducted using a leave-one-out meta-analysis. Publication bias analysis was conducted using Begg’s and Egger’s tests, as well as a funnel plot.

**Results:**

In total, 11 studies involving 130,079 participants were included in the meta-analysis of the association between low lean mass and the risk of all-cause mortality in the middle-aged and older population, all of which the included studies were of high quality. The average overall study quality score was 8 points. The random effects model analysis results showed that the pooled RR of all-cause mortality risk in the middle-aged and older population was 1.30 (95% CI, 1.16–1.47, *P* < 0.001) across the lowest to normal lean mass category. There was an inverse non-linear dose-response relationship between lean mass and the risk of all-cause mortality (*P* < 0.001).

**Conclusion:**

Low lean mass was significantly associated with 30% higher risk of all-cause mortality in the middle-aged and older population. These findings highlighted low lean mass as an important risk factor for mortality in middle-aged and older population, warranting its integration into clinical assessments. Future research should establish causality through longitudinal studies and randomized trials, while refining diagnostic cutoffs for diverse populations.

**Systematic review registration:**

https://www.crd.york.ac.uk/PROSPERO/#myprospero, Identifier CRD42023445297.

## Introduction

The global trend of aging is irreversible, leading to an increasing economic and medical burden (1). Therefore, promoting healthy aging in the middle-aged and older population has become an urgent priority in public health. The association between the body tissue composition in the middle-aged and older population and the risk of all-cause mortality has always been a topic of controversy (2). Typically, lean mass is equivalent to muscular mass. The body composition of older populations changes with aging (3). For example, as individuals get older, there is a progressive decrease in lean body mass, including appendicular lean mass and skeletal muscle index (4, 5). As reported by studies, the average yearly percentage of muscle decline in individuals over 50 years was 1–2% (6). Nevertheless, aging would not only hasten lean mass loss but also promote body fat accumulation, resulting in an increase in the quantity of fat in internal organs (7). There is mounting evidence that obesity, as measured by body mass index (BMI), is an important factor contributing to the increased death risk of people (8, 9). However, an overweight or higher BMI value may be protective against all-cause mortality in older people when compared to a normal BMI, and this phenomenon is known as the “obesity paradox”, where higher BMI sometimes correlates with lower all-cause mortality rates (10, 11).

The BMI is not a precise indicator of obesity, as it is unable to distinguish between lean mass and fat mass (12). This is particularly essential given that individuals with the same BMI had vastly different body compositions. Different amounts of fat mass and lean mass may have opposing impacts on health (13). To correctly interpret the “obesity paradox”, it might be necessary to investigate separately the effects of lean mass and fat mass on the risk of all-cause mortality. Some recent studies have revealed that lean mass is an important predictor of physical function and mortality (14) in older people. Furthermore, a systematic review and meta-analysis of 35 prospective cohort studies involving 923,295 participants showed that excessive fat was detrimental to health and a higher body fat percentage was associated with a higher risk of all-cause mortality in a J-shaped manner (15). Due to the difficulty and high cost of detecting lean mass, particularly in large cohort studies of the middle-aged and older individuals with extended follow-up, little is known about the effect of lean mass on death risk (16).

Thus, the purpose of this study is to collect prospective cohort studies to conduct a meta-analysis of the effect of low lean mass on all-cause mortality risk in the middle-aged and older population and to provide valuable clinical and public health information about healthy body composition.

### Methods

The registration number for this study on PROSPERO is CRD42023445297. This meta-analysis was reported using the Preferred Reporting Items for Systematic Reviews and Meta-Analyses (PRISMA) 2020 guidelines (17).

### Search strategy

The PubMed, Web of Science, and Scopus databases were searched until May 22, 2025, for relevant publications. The search methodology was described in detail in Supplementary Table 1, and we also searched references to relevant literature to ensure a comprehensive search.

### Study selection

During the initial search, two authors (JL and XLL) systematically assessed the titles and abstracts of all eligible articles before reviewing the full text. The third author (YHW) resolved disputes through arbitration in order to achieve a consensus. We investigated prospective cohort studies (the baseline population is healthy) assessing the association between low lean mass and the risk of all-cause mortality in the middle-aged and older population. The inclusion criteria for this review were as outlined below: (1) The study employed a prospective cohort design, with a focus on low lean mass as the exposure of interest; (2) The primary outcome measure of interest was the risk of all-cause mortality; (3) The risks of all-cause mortality were reported using relative risk (RR), hazard ratio (HR), or odds ratio (OR), collectively with their accompanying 95% confidence intervals (CIs). In the interim, the following exclusion criteria applied: (1) Participants were not selected from a population that is generally in good health; (2) Excluded from consideration were papers that met the following criteria: reviews, randomized controlled trials (RCTs), case-control studies, retrospective cohort studies, non-human studies, non-English studies, and letters missing adequate data; (3) participants were not recruited from an middle-aged and older population (age under 45 years old). Only the reports with the longest follow-up and biggest sample size were included in cases where multiple reports from the same study were available.

### Outcome

The outcome was all-cause mortality.

### Assessment of low lean mass

According to previous literature, we defined appendicular lean mass as the weight of skeletal muscle in the limbs after removing fat (18), lean body mass as the weight typically after removing body fat (19), and lean mass as the weight of skeletal muscle after removing body fat (4). In this study, we defined low lean mass as the loss of muscle mass due to aging or any underlying disease, regardless of whether muscle function declines or adipose tissue is depleted, including one of the three indicators mentioned above.

### Data extraction

Two trained researchers are responsible for the data extraction process (JL and XLL). Each study that met the inclusion criteria had its data extracted onto a standardized form that sought the following information: first author’s last name, year of publication, study design, location, sample size (total sample/number of deaths), mean age, number of years of follow-up, participants’ BMI, method of lean mass assessment, and indicators reported for low lean mass (lean mass or lean body mass, or appendicular lean mass). If studies presented data separately by gender, the results would be analyzed as two independent reports.

### Quality assessment

All included studies were evaluated by two trained researchers (JL and XLL) using the Newcastle-Ottawa Scale (NOS) for quality. The Cochrane Collaboration recommended the NOS as a risk of bias assessment instrument for observational studies (20). The NOS allocates a maximum of nine points for the lowest level of bias in three domains: (a) the process of selection of study groups (four points); (b) comparability of groups (two points); and (c) ascertainment of exposure and outcomes (three points) for case–control and cohort studies, respectively (20). The higher the research scores, the higher the quality of the study. We determined that NOS scores of 0–3, 4–6, and 7–9 were of low, medium, and high quality, respectively. The quality of the evidence used to support the outcomes was assessed using the Grading of Recommendations, Assessment, Development, and Evaluation (GRADE) system (21). As an example, observational studies were given a poor starting quality rating under the GRADE guideline, but this rating might go up or down depending on other parameters. Disagreements were resolved via dialogue with the third reviewer (YHW).

### Statistical analysis

The study used a random-effects model to combine risk estimates with 95 percent confidence intervals in order to assess the risks of all-cause mortality between individuals with low lean mass and those with normal lean mass (reference), producing more conservative results than a model with a fixed effect. According to the Cochrane Handbook for Systematic Reviews of Interventions Version 5.1.0, RR and HR were approximately equivalent (22). In the meantime, an OR was converted to an RR using the following formula: RR = OR/[(1-P_0_) + (P_0_OR)], where P_0_ represents the reference group’s death rate (23). Using the Q test and the I^2^ statistic, we evaluated the heterogeneity between studies (24). A *P*-value less than 0.05 in the Q test or an I^2^ greater than 50% indicated the presence of significant heterogeneity.

To investigate potential sources of between-study heterogeneity, subgroup analyses were carried out. These analyses focused on factors such as age at baseline, gender, BMI at baseline, location of study, duration of follow-up, number of participants, method used for assessment of lean mass, study quality, and indicators reported for lean mass. Additionally, we conducted a meta-regression model analysis based on participants’ age, follow-up time, region, gender, and sample size.

Studies reported at least three category groups of lean mass with the same indicator were included in dose-response analyses, where the lowest category of lean mass was specified as a reference. For studies with a non-lowest class reference, the method proposed by Hamling et al. is used for the estimated transformation (25). Possible non-linear dose-response relationships between lean mass and all-cause mortality were examined through a random-effect dose-response analysis using a restricted cubic spline model with three nodes at the 10th, 50th, and 90th percentiles of the distribution, using a likelihood ratio test to assess the difference between linear and non-linear models (25). Since the association for predicted lean mass is approximately linear, we separately used a linear model to calculate the pooled RR per unit (kg) of lean mass gain.

Sensitivity analysis was conducted using a leave-one-out meta-analysis (LOOM)., i.e., removing one piece of research at a time to assess the robustness of the primary results and the impact of each report on the effect or heterogeneity. Evaluation of publication bias using funnel plots, Begg’s and Egger’s tests. A *P*-value less than 0.10 indicates the existence of publication bias (26). The trim and fill method was used wherever there was evidence of publication bias (27).

We used STATA version 16.0 (Stata Corp., College Station, TX, United States) to run data studies, and we put the data in twice to avoid making mistakes. A *P*-value of less than 0.05 was considered statistically significant unless otherwise stated.

## Results

### Literature screening process

**FIGURE 1:**
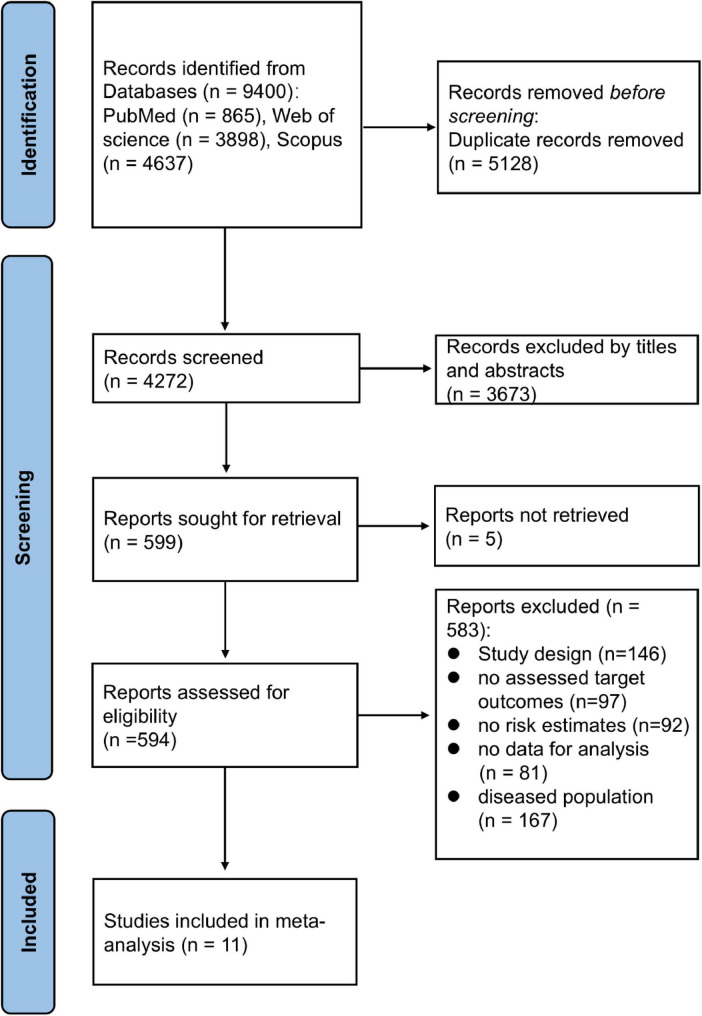
Flow chart of study selection.

Figure 1 shows the flowchart illustrating the selection process for the study. Out of 9,400 initial articles, 594 were selected for full-text examination after removing duplicates (*N* = 5,128) and screening titles and abstracts (*N* = 3,673), and 583 articles were removed for the following reasons: report not retrieved (*N* = 5); study design was inconsistent (*N* = 146); no risk estimates (*N* = 92); no assessed target outcomes (*N* = 97); no sufficient data for qualitative analysis (*N* = 81); and diseased population (*N* = 167). Ultimately, 11 studies (14, 16, 28–36) were included in the final meta-analysis.

### Characteristics of included studies

Eleven articles (14, 16, 28–36) with fourteen reports reported the association between low lean mass and all-cause mortality risk in the middle-aged and older population. There was a total of 130,079 participants in eight studies, of whom 31,158 died during the follow-up period. The minimum sample size was 715 (35), the maximum sample size was 55,818 (14), and the average age of the participants ranged from 45 to 84.2 years. One study was performed in France (35), and ten studies were conducted in the United States (16, 28–31, 33, 34, 36). The study with the shortest duration of research lasted 3.3 years (29), and the largest lasted 21.4 years (16). Only one of the 11 studies utilized the bioelectrical impedance (BIA) (30) method to detect low lean mass, and the remaining ten utilized the dual x-ray absorptiometry (DXA) (14, 16, 28, 29, 31–36) method. Four studies examined appendicular lean mass (skeletal muscle weight after removing fat from limbs) indicators (29, 33, 34, 36), three studies examined lean body mass (the weight of the human body after removing fat) indicators (16, 28, 35), and four studies examined lean mass (skeletal muscle weight after removing fat in the human body) (14, 30–32) (Table 1).

**TABLE 1:** Characteristics of included studies for all-cause mortality (11 studies).

References	Country	Study name	N (deaths)	Mean age (years)	Duration	Exposure assessment	Muscle categories	Corresponding relative risk (95% CI)	References
Bea et al. (28)	USA	Women’s Health initiative 1993–1998	10,525 (1,762)	63.1	13.6 (4.6)	DXA	Lean body mass,%: Q1 (≤ 47.2) Q2 (47.3–51.1) Q3 (51.2–54.5) Q4 (54.6–58.7) Q5 (≥ 58.8)	All-cause mortality: 1 1.02 (0.85, 1.21) 0.89 (0.75, 1.07) 0.98 (0.82, 1.17) 1.01 (0.84, 1.21)	(28)
Cawthon et al. (29)	USA	Osteoporotic fractures in men 2014–2016	1,400 (197)	84.2	3.3	DXA	ALM, kg: q1 q2 q3 q4	All-cause mortality: 1.6 (0.7, 3.8) 1.2 (0.6, 2.4) 1.4 (0.8, 2.5) 1	(29)
Farsijani et al. (31)	USA	Health ABC study	Male: 1,414 (1,003) Female: 1,497 (870)	Male: 73.8 Female: 73.5	12(5)	DXA	Lean mass (per 9.9kg decrease): Male Female	All-cause mortality: 1.32 (0.61, 2.86) 1.23 (0.47, 3.23)	(31)
Liu et al. (32)	USA	The national Health and nutrition examination survey (2003–2006)	5,052 (826)	45	14.6	DXA	Lean mass, kg: Q1 Q2 Q3 Q4 Q5	All-cause mortality: 1 0.56 (0.42, 0.76) 0.50 (0.37, 0.67) 0.47 (0.33, 0.67) 0.34 (0.23, 0.50)	(32)
Lee et al. (16)	USA	Health Professionals Follow-up Study 1987–2012	38,006 (12,356)	54.4	21.4	Anthropometry	Lean body mass, kg: 5th 35th 50th 65th 95th	All-cause mortality: 1 0.92 (0.87, 0.97) 0.90 (0.85, 0.96) 0.92 (0.87, 0.98) 0.97 (0.91, 1.04)	(16)
Liu et al. (14)	USA	Nhanes study (1988–1994 and 1999–2014)	55,818 (10,408)	45	9.7	DXA	Lean mass, kg: Q1 Q2 Q3 Q4 Q5	All-cause mortality: 1.64 (1.46, 1.83) 1.29 (1.18, 1.42) 1 0.95 (0.87, 1.04) 0.88 (0.75, 1.03)	(14)
Dolan et al. (30)	USA	Women aged 65y and older	8,029 (945)	73.6	8	BIA	Lean mass, kg: Q1 Q2 Q3 Q4 Q5	All-cause mortality: 1 0.88 (0.72, 1.08) 0.83 (0.67, 1.08) 0.88 (0.70, 1.09) 1.16 (0.92, 1.45)	(30)
McLean et al. (33)	USA	Foundation for the national institutes of health sarcopenia Project	Male: 4,411 (19) Female: 1,869 (18)	Male: 74.0 Female: 76.5	8.4	DXA	ALM, kg: Male: < 19.75 Female: < 15.02	All-cause mortality: 1.37 (1.03,1.82) 1.07 (0.81,1.41)	(33)
Santanasto et al. (34)	USA	Health ABC Study (participants had both a baseline (1997–1998) and a valid 2002–2003 CT scan)	Male: 869 (528) Female: 934 (467)	Male: 78.5 Female: 78.1	11.5 ± 0.9	DXA	ALM (per 1.2 kg decrease): Male Female	All-cause mortality: 1.10 (0.98, 1.23) 1.06 (0.91, 1.24)	(34)
Szulc et al. (35)	France	MINOS study	715 (137)	65.0	10	DXA	Low Lean body mass	All-cause mortality: 2.78 (1.38, 5.57)	(35)
Wang et al. (36)	USA	NHANES study (1999–2,002)	2,540 (1,615)	70.43	13.15	DXA	ALM, kg Male: < 19.75kg Female: < 15.02kg	All-cause mortality: 1.46 (1.45, 1.46)	(36)

ALM, appendicular lean mass; BIA, bioelectrical impedance; BMI, body mass index; DXA, dual x-ray absorptiometry; q, quartile; Q, quinti; NHANES, The National Health and Nutrition Examination Survey.

### Study quality

The NOS was used for assessing the study’s quality, and the scores were presented in Table 2. Based on the NOS score, each of the included studies was of high quality. The average overall study quality score was 8 points.

**TABLE 2:** Study quality of studies included in the analysis assessed by the Newcastle Ottawa Scale.

References	Selection	Comparability	Outcome	Total score
Bea et al. (28)	3	2	3	8
Cawthon et al. (29)	3	2	2	7
Farsijani et al. (31)	4	2	3	9
Liu et al. (14)	4	2	3	9
Liu et al. (32)	3	2	3	8
Lee et al. (16)	2	2	3	7
Dolan et al. (30)	3	2	3	8
McLean et al. (33)	3	2	3	8
Santanasto et al. (34)	4	2	2	8
Szulc et al. (35)	3	2	3	8
Wang et al. (36)	3	2	3	8

Selection: (1) Representativeness of the exposed cohort; (2) Selection of the non-exposed cohort; (3) Ascertainment of exposure; (4) Demonstration that outcome of interest was not present at start of study; Comparability: (1a) study controls for age (the most important factor); (1b) study controls for any additional factor; Outcome: (1) Assessment of outcome; (2) Was follow-up long enough (≥ 5 years) for outcomes to occur; (3) Adequacy of follow up of cohorts (≥ 80%).

### Low lean mass and all-cause mortality risk

**FIGURE 2:**
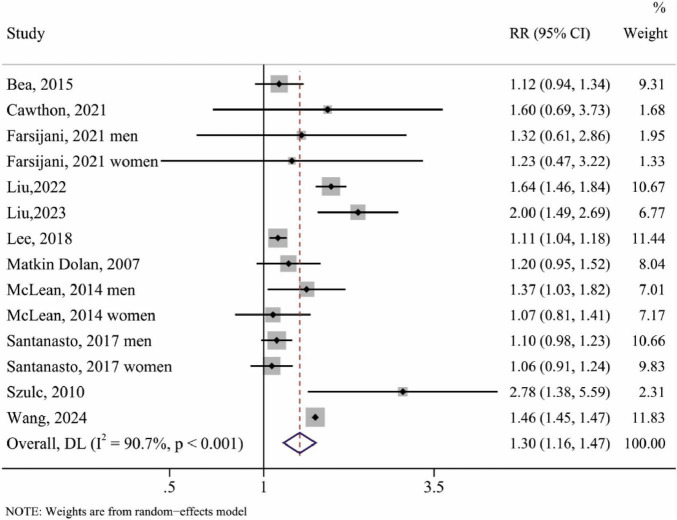
The forest plot of low lean mass (lowest vs. normal category of lean mass) and the risk of all-cause mortality.

In total, 11 studies with 14 reports were included in the meta-analysis of the association between low lean mass and the risk of all-cause mortality in the middle-aged and older population. Analysis of the random-effects model showed that the pooled RR for the risk of all-cause mortality in the middle-aged and older population was 1.30 (95% CI, 1.16–1.47, *P* < 0.001) in the lowest to normal lean mass category, and the result indicated a 30% higher risk of all-cause mortality for individuals with low lean mass compared to those with normal lean mass (Figure 2 and Table 3).

**TABLE 3:** Subgroup analysis of low lean mass and risk of all-cause mortality.

Variables	n	RR (95% CI)	*P* ^1^	Heterogeneity		Meta-regression
				I^2^ (%)	*P* ^2^	*P* ^3^
**All-cause mortality**	14	**1.30 (1.16–1.47)**	**<0.001**	90.7	**<0.001**	
**Age at baseline**						**0.041**
45-65 years	4	**1.39 (1.08–1.81)**	**<0.001**	93.5	**<0.001**	
65-75 years	6	**1.42 (1.27–1.58)**	**<0.001**	20.8	0.277	
75-85 years	4	**1.08 (0.99–1.19)**	**0.054**	0.0	0.812	
**Gender**						0.761
Male	6	**1.18 (1.05–1.34)**	**0.007**	47.1	0.092	
Female	5	**1.10 (1.00–1.22)**	**0.048**	0.0	0.929	
**Indicator**						0.124
Lean mass	5	**1.54 (1.26–1.88)**	**<0.001**	53.1	0.074	
Lean body mass	3	1.20 (0.97–1.47)	0.093	69.7	**0.037**	
Appendicular lean mass	6	**1.22 (1.02–1.46)**	**0.032**	89.0	**<0.001**	
**Country**						0.076
France	1	**2.78 (1.38–5.59)**	**<0.001**	0.0	–	
USA	13	**1.28 (1.14–1.44)**	**0.004**	91.2	**<0.001**	
**Follow-up years**						0.435
<10	6	**1.41 (0.28–1.57)**	**<0.001**	**57.6**	**0.038**	
≥ 10	8	**1.21 (1.07–1.37)**	**0.003**	68.1	**0.003**	
**No. participants**						0.554
< 5000	7	**1.30 (1.06–1.60)**	**0.013**	86.3	**<0.001**	
≥ 5000	7	**1.31 (1.10–1.57)**	**0.002**	87.5	**<0.001**	
**Detection**						0.703
DXA	13	**1.31 (1.16–1.49)**	**<0.001**	91.3	**<0.001**	
BIA	1	1.20 (0.95–1.52)	0.129	0.0	–	

P^1^value for RR; P^2^ value for heterogeneity between studies; P^3^ value for meta-regression; significant *p* values are highlighted in bold prints. BMI, body mass index; BIA, bioelectrical impedance; DXA, dual x-ray absorptiometry.

### Subgroup analysis and meta-regression

The results of subgroup analysis and meta-regression were shown in Table 3. Subgroup analyses showed low lean mass was significantly associated with an increased risk of all-cause mortality in males (RR: 1.18, 95% CI, 1.05–1.34, *P* = 0.007), while the correlation was weak in females (RR: 1.10, 95% CI, 1.00–1.22, *P* = 0.048). Moreover, both lean mass (RR: 1.54, 95% CI, 1.26–1.88, *P* < 0.001) and appendicular lean body mass (RR: 1.22, 95% CI, 1.02–1.46, *P* = 0.032) were significantly associated with an increased risk of all-cause mortality, but it was not observed in lean body mass (RR: 1.20, 95% CI, 0.97–1.47, *P* = 0.093). The significant association was observed only in lean mass assessed by dual x-ray absorptiometry (DXA, RR: 1.31 95% CI, 1.16–1.49, *P* < 0.001), but not in that assessed by bioelectrical impedance (BIA, RR: 1.20 95% CI, 0.95–1.52, *P* = 0.129). The significant association was observed in age of 45–65 years (RR: 1.39 95% CI, 1.08–1.81, *P* < 0.001) and age of 65–75 years (RR: 1.42 95% CI, 1.27–1.58, *P* < 0.001), but not in age of 75–85 years (RR: 1.08 95% CI, 0.99–1.19, *P* = 0.054).

**FIGURE 3:**
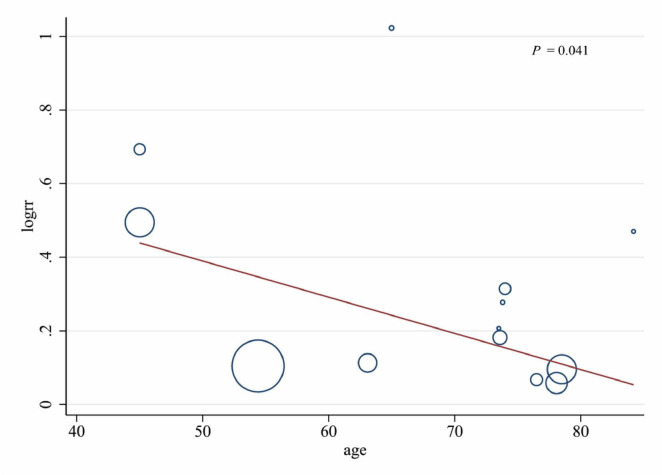
Meta-regression analysis of low lean mass (lowest vs. normal category of lean mass) and all-cause mortality by age.

The results of the meta-regression analysis showed that the average age of participants (*P* = 0.041) was the influential factor for low lean mass being significantly associated with an increased risk of all-cause mortality in the middle-aged and older population. A significant negative association between participants’ age and the low lean mass-associated mortality was observed (Figure 3). Through subgroup analysis and regression analysis, we found that the sample size of participants, the detection method of low lean mass, and the age of participants were potential sources of high heterogeneity.

### Dose-response analysis

**FIGURE 4:**
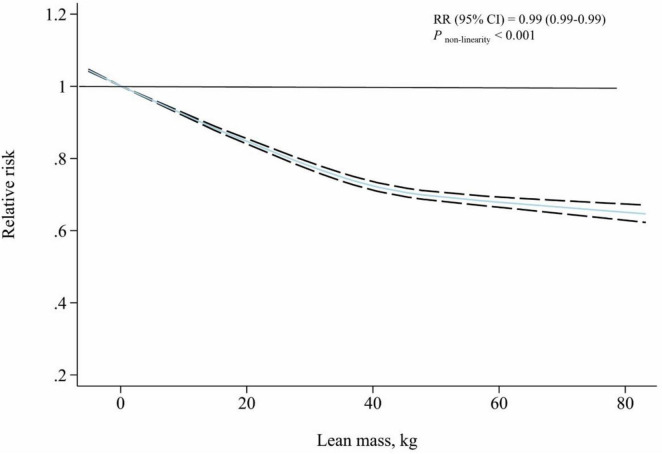
The association between lean mass and all-cause mortality risk for dose-response analysis.

Out of all 11 studies, four studies (14, 30–32) were eligible for the lean mass and all-cause mortality risk dose-response relationship analysis, including 106,905 subjects and 24,535 cases. A significant inverse association between lean mass and all-cause mortality risk was found in the non-linear dose-response analysis (*P*_*non–linearity*_ < 0.001, 4 studies, Figure 4). The estimates of trend effect showed a 1% reduction in the risk of all-cause mortality for every 1 kg increase in lean mass (RR: 0.99 95% CI, 0.99–0.99, *P* < 0.001, Table 4).

**TABLE 4:** The result of model selection of dose-response analysis.

Result	χ^2^	*P* (likelihood ratio)	Effect size (95% CI)	Model select	*P* (model)	Relationship
**Lean mass, kg**	1,658.5	**<0.001**	0.99 (0.99–0.99)	Non-linear	**<0.001**	Inverse

Significant *p*-values are highlighted in bold prints.

### Sensitivity analysis

**FIGURE 5:**
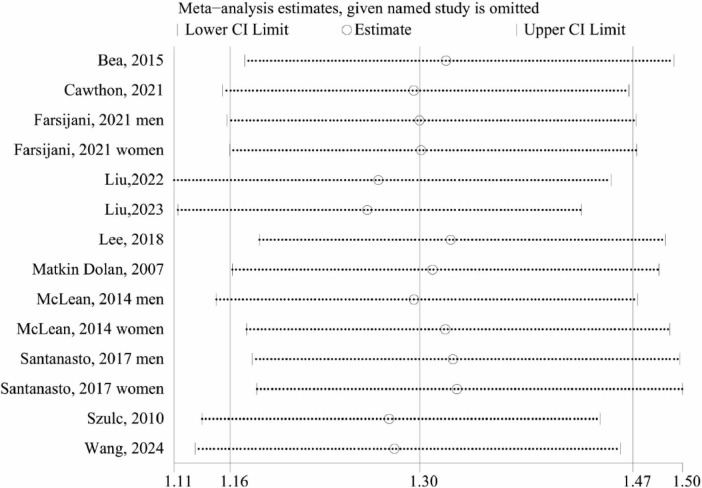
Sensitive analysis of low lean mass (lowest vs. normal category of lean mass) and all-cause mortality.

We carried out LOOM analysis for sensitivity to assess the robustness of the overall effect size. The sensitivity analysis results demonstrated the robustness of our findings, confirming that the association between low lean mass and increased all-cause mortality risk remains strong even when individual studies are excluded. (Figure 5).

### Publication bias

**FIGURE 6:**
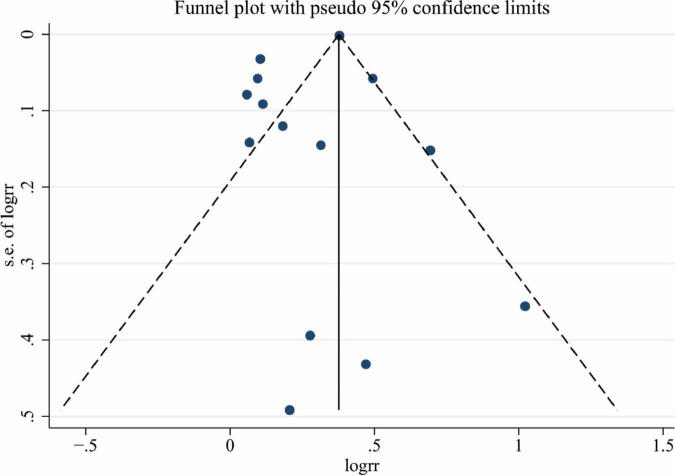
The result of the funnel plot for publication bias.

Finally, we conducted a publication bias test, the funnel plot, Begg’s test, and Egger’s test all revealed that there was no publication bias in this study (*P* = 0.113, 0.120, respectively) (Figure 6).

## Discussion

This meta-analysis demonstrates a significant association between low lean mass and increased mortality risk in the middle-aged and older population. We found a statistically significant association between low lean mass and an increased risk of all-cause mortality in the middle-aged and older population compared to a control group with normal lean mass. Subgroup analyses revealed that the significant association between low lean mass and the increased risk of all-cause mortality might be influenced by indicators of lean mass and detection method of lean mass The results of meta-regression showed that the heterogeneity among studies might be due to the differences in the age of participants. Dose-response analysis showed a significant inverse non-linear association between lean mass and all-cause mortality risk.

Consistent with our findings, previous studies have also shown a substantial negative correlation between low lean mass and increased all-cause mortality risk (37). To the best of our knowledge, our study is the first to demonstrate an inverse non-linear dose-response relationship between lean mass and all-cause mortality, further suggested that increasing lean mass may reduce the risk of all-cause mortality. Furthermore, although this study found that for every 1 kg increase in lean body mass, the mortality rate decreases by 1%, the clinical significance of this finding deserves careful interpretation. Due to aging and disease, elderly people naturally lose a certain amount of lean body mass each year, and interventions such as resistance training or nutritional strategies can only partially alleviate this loss. However, in populations with higher baseline mortality rates, such as elderly frail individuals, this absolute risk reduction level may bring meaningful clinical benefits. The health and longevity of the older population were significantly influenced by lean mass, including lean mass and appendicular lean mass (38). Lean body mass (including visceral and trunk muscles) is the main determinant of basal metabolic rate (39). Appendicular lean mass is the core manifestation of skeletal muscle function, directly reflecting limb movement ability (40). Variations in the proportion of visceral muscles may influence the strength of the relationship between lean mass and various outcomes. Consequently, discrepancies in the definition of lean mass represent a source of heterogeneity. To mitigate bias, future research should implement standardized protocols for measuring lean mass. Lean mass was a diagnostic marker for numerous aging-related disorders, including sarcopenia, muscular dystrophy, cancer, and so on (41–43). Given the inevitable decline in muscle strength that occurs with aging, a higher lean mass was also required to maintain normal life and physical function in the middle-aged and older population (44). Lean mass is the key tissue of body activity, and low lean mass has a negative impact on daily life, reducing the ability to perform daily activities and prolonging the recovery time from disease. Among lean mass, skeletal muscle is the main energy metabolism tissue, which participates in the uptake, utilization and storage of energy metabolism substrates such as glucose and amino acids (45). Low lean body mass is also associated with decreased muscle strength and functional capacity, increasing fall and fracture risks. Additionally, the loss of lean mass can directly lead to a decrease in metabolic rate, increase the risk of obesity, insulin resistance, and a variety of comorbidities (46), ultimately increase the risk of death (4). Physical exercise has been considered an effective intervention for increasing lean mass in the middle-aged and older population. Physical exercise could not only reduce the older population’s body fat but also enhance their muscle protein synthesis function, thereby increasing their lean mass (47).

Our study found that low lean mass has a stronger negative association with all-cause mortality in middle-aged adults than in the older adults. Anabolic resistance and lack of exercise are major drivers of age-related muscle loss. Therefore, the older population are more susceptible to muscle wasting. It has been reported that age-related muscle mass loss accounts for 42% of muscle mass and declines rapidly after 50 years of age (48). Our results also suggested that, interventions for the remediation and prevention of age-related muscle loss should begin as early as possible, when muscle atrophy is most severe. In addition, uncorrected hydration status in BIA studies may lead to underestimation of lean body mass in the limbs, which may weaken its association with outcomes. Future studies should give priority to using DXA or multi-frequency BIA calibrated for the older population, and combine functional indicators (such as muscle strength) to enhance the clinical relevance of body composition assessment.

According to our findings, low lean mass in male studies has a higher risk of all-cause mortality in the middle-aged and older population people than in female studies. After analyzing the included studies, we found that the average age for male studies was 71.65 years old and for female studies was 72.96 years old. Some studies have pointed out that the disease burden of older men was higher than that of older women (49, 50), and in terms of all-cause mortality and disability by gender, annual global deaths and DALYs among men were approximately 15% higher than for women (51), which could partially explain the results of our study. In general, the most widely utilized techniques for measuring lean mass were BIA and DXA. BIA was regarded as the least appropriate detection method (52), even though both methods of detecting lean mass have been demonstrated to be significantly connected with an elevated risk of all-cause mortality (53). This is mostly because there were several factors that can affect the BIA results, such as no standardization of body position, previous physical activity, and food or fluid intake, and the accuracy of BIA measurements decreases when specific prediction equations and standardized measurement protocols are not used, as they are unable to distinguish between intracellular and extracellular water (54). The validity of BIA measurements in older age groups may be impacted by hydrostatic disturbances, peripheral edema, and the use of diuretic medication (55). Aging-related changes in hydration have been linked to a decline in total body water and fat-free mass that comes with getting older (56). Additionally, a study found that BIA techniques that had been verified for the prediction of lean mass in young people were insufficient when applied to the older population (57). It further explained our findings that the low lean mass of the DXA-based detection method is significantly associated with an increased risk of death from all causes in the older population, which is not reflected via the BIA detection method. Future research should attempt to standardize lean mass testing methods and indicators, prioritizing the use of multi-sites CT/MRI to quantify lean mass, and standardizing processes to reduce confusion, and conducting large-scale cohorts to clarify this association.

### Strengths and limitations

This meta-analysis still has some limitations. Although we only included a healthy middle-aged and older population in the study sample, the heterogeneity of the study was also very high. Our subgroup analysis results were still limited by the inclusion of fewer studies. Additionally, there are differences in the definition and adjustment methods of low lean body mass and confounding factors included in the study, which may introduce residual heterogeneity. Future research needs to adopt a standardized definition of lean body mass and systematically control for covariates such as physical activity and comorbidities. Besides, 10 of 10 included studies originate from the United States, which constrains the global applicability of our findings. Due to design and data limitations, the current study cannot determine the duration of low body weight required for adverse reactions and whether they can be reversed through treatment, and this study was limited to examining all-cause mortality; future investigations with larger sample sizes should evaluate cause-specific mortality, particularly cardiovascular and cancer-related deaths, to better understand the differential impacts of low lean mass. Finally, we hope that in the future, more and more cohort studies can focus on how to explore the relationship between lean mass and mortality in diverse populations.

## Conclusions

Compared with the normal lean mass group, individuals with low lean mass had a 30% higher risk of all-cause mortality risk. These findings highlighted low lean mass as an important risk factor for mortality in middle-aged and older population, warranting its integration into clinical assessments. Future research should establish causality through longitudinal studies and randomized trials, while refining diagnostic cutoffs for diverse populations.

## Data Availability

The datasets presented in this study can be found in online repositories. The names of the repository/repositories and accession number(s) can be found in the article/Supplementary material.
