# Supplementary material for: Low lean mass and all-cause mortality risk in the middle-aged and older population: a dose-response meta-analysis of prospective cohort studies
Source: Front Med (Lausanne). 2025 Jun 25;12:1589888. doi: 10.3389/fmed.2025.1589888 (PMC12237975; doi:10.3389/fmed.2025.1589888)
Supplement: Supplementary file 1 [file Table_1.docx]

**Table SI: Search strategy**

| Example search strategy in PubMed. |
| --- |
| ((“Lean mass” [Title/Abstract]) OR (“lean body mass” [Title/Abstract]) OR (“Appendicular lean mass ” [Title/Abstract]) OR (“Fat Free Mass ” [Title/Abstract])) AND ((death [Title/Abstract]) OR (“death rate” [Title/Abstract]) OR (mortality [Title/Abstract]) OR (mortalities [Title/Abstract]) OR (fatal [Title/Abstract]) OR (survival [Title/Abstract]) OR (deceased [Title/Abstract]) OR (“survival rate” [Title/Abstract])) AND ((cohort studies [Mesh]) OR cohort OR prospective OR follow-up) NOT ((“comment”[Publication Type]) OR (“letter”[Publication Type])) |
| Example search strategy in Web of science and Scopus |
| ((“Lean mass”) OR (“lean body mass”) OR (“Low lean body mass”) OR (“Low lean mass”) OR (“Appendicular lean mass”) OR (sarcopenia) OR (Fat Free Mass)) AND ((death) OR (“death rate”) OR (mortality) OR (mortalities) OR (fatal) OR (survival) OR (deceased) OR (“survival rate”)) AND (“cohort studies” OR cohort OR prospective OR follow-up) |
